# Role of Vitamin E in Neonatal Neuroprotection: A Comprehensive Narrative Review

**DOI:** 10.3390/life12071083

**Published:** 2022-07-20

**Authors:** Sarah Kolnik, Thomas R. Wood

**Affiliations:** 1 Division of Neonatology, Department of Pediatrics, University of Washington, Seattle, WA 98195, USA; tommyrw@uw.edu; 2 Center on Human Development and Disability, University of Washington, Seattle, WA 98195, USA

**Keywords:** vitamin E, neuroprotection, neonatal, hypoxia-ischemia

## Abstract

Vitamin E (Vit E) is an essential lipophilic antioxidant and anti-inflammatory agent that has potential as a neuroprotectant in newborn infants with brain injury. Vit E has shown promise in many in vitro studies, but success in translation to in vivo animal studies and the clinical setting has been mixed, with concern of adverse effects at high intravenous doses in preterm infants. However, a recent rise in knowledge of the beneficial effects of fat emulsions containing higher levels of Vit E, along with associated improved outcomes in some neonatal co-morbidities, has led many to reconsider Vit E administration as a potential therapeutic modality to improve neurological outcomes in the setting of neonatal brain injury. This narrative review discusses Vit E’s structure, mechanism(s) of action, evidence in animal models, and association with health outcomes in neonates, including both dietary and supplemental Vit E and their bioavailability and pharmacokinetics as it relates to the brain. Lastly, long-term neurodevelopmental outcomes along with gaps in current knowledge are critiqued, which to date suggests that additional translational studies in larger animal models and assessment of safety profiles of different routes and doses of administration should be explored prior to large clinical trials. Importantly, a greater understanding of the brain region(s) and cell type(s) affected by Vit E may help to target the use of Vit E as a beneficial neuroprotective agent to specific populations or types of injury seen in newborns.

## 1. Introduction

Vitamin E (Vit E; α-, β-, γ-, and δ-tocopherol and α-, β-, γ-, and δ-tocotrienols) is an essential lipophilic antioxidant and anti-inflammatory agent that has potential neuroprotective properties [1,2]. Neonatal neurological injury can manifest or be exacerbated by oxidative stress and inflammation; therefore, finding a viable treatment to dampen these delirious effects has led many to examine Vit E as a potential neuroprotective agent in newborn infants with brain injury [3]. Furthermore, Vit E deficiency results in maldevelopment of the nervous system as Vit E is essential for normal embryonic development, neurogenesis, and cognition [4]. However, to date, the evidence supporting the use of Vit E as a neuroprotectant is mixed. Vit E has been investigated in many rodent models, but these have varying efficacy when translated clinically [5,6]. In very low birth weight (VLBW) infants, clinical studies using high dose intravenous Vit E have also reported adverse effects [5]. In contrast, more recent positive evidence of Vit E as a neuroprotective agent in a term in vitro ferret model of hypoxic ischemic brain injury suggests promise [7]. As advances in neonatal care are improving rates of mortality, it is imperative that we continue to probe new therapeutic modalities to combat neurological injury in the neonatal period [8]. The aim of this review is therefore to compile a comprehensive overview of Vit E as it relates to neuroprotection in newborns.

## 2. Structure and Isoforms

Vit E exists in nature as isoforms of tocopherol and tocotrienol. There are alpha (α), beta (β), gamma (γ), and delta (Δ) isoforms of both tocopherol and tocotrienol, resulting in eight total Vit E chemical forms [9,10,11]. The most biologically active form is α-tocopherol, which in its natural configuration of D-α-tocopherol (RRR- α-tocopherol) is susceptible to oxidation; therefore, when made synthetically, the phenol group is converted to an ester, making the configuration DL- α-tocopherol (all racemic α-tocopherol), which is more stable [9]. However, the natural D-form of Vit E is more bioactive than racemic synthetic form [12,13]. The enhanced activity of α-tocopherol is thought to be due, in part, to α-tocopherol transfer protein (α-TTP), a cytosolic transport protein that facilitates selective enrichment of α-tocopherol by transferring it into plasma lipoproteins [14]. Recent evidence shows α-TTP expressing astrocytes control delivery of Vit E from astrocytes to neurons and that this process is responsive to oxidative stress [15]. Although both tocopherols and tocotrienols have antioxidant capacity, only α-tocopherol is retained, as the other tocopherols and tocotrienols are excreted after reaching the liver in remnant chylomicrons [16]. Interestingly, the type of α-tocopherol used in formula supplementation may also alter the brain’s uptake and storage. For example, in a recent study of neonatal primates exposed to formula supplemented with either naturally or synthetically derived α-tocopherol demonstrated a 1.5x fold increase in the more biologically active form of Vit E, RRR- α-tocopherol, in brain tissue in those fed naturally compared to synthetically derived α-tocopherol [12]. Of the other Vit E isoforms, γ-tocopherol is the most abundant in the western diet as it is present in soy and corn oil, but it is not biologically active [13]. By comparison, α-tocopherol is predominately found in peanut, almond, and sunflower oils, and is dietarily essential [11]. Humans can absorb all forms of Vit E but cannot interconvert them; therefore, only α-tocopherol is thought to contribute to Vit E demands in humans [10]. As a result, measurements of neonatal levels and supplementation generally refer only to biologically active α-tocopherol [5,6,10].

## 3. Vit E Absorption and Pharmacokinetics

In order to be absorbed from the gut, Vit E and lipids must first form micelles in the presence of bile salts. Once this step is complete, Vit E is absorbed via passive diffusion in the small intestine [17]. For adequate absorption, neonates must therefore have intact pancreatic function and adequate bile salt excretion and fat intake during enteral Vit E administration [17]. Vit E is eliminated via fecal excretion or conjugation. As noted above, α-tocopherol is the most bioavailable, and other forms of Vit E cannot be transformed by the body. Furthermore, in pediatric patients with chronic cholestasis and thus poor absorption of lipid soluable Vit E, comparable dosing of D-alpha-tocopheryl polyethylene glycol 1000 succinate (TPGS1000), an aqueous solution, has been successful in safetly optimizing Vit E levels [18,19]. Intravenous dosing bypasses any challenges to enteric absorption; however, clinical studies suggest toxicity of Vit E at high doses intravenously, showing associations with sepsis and necrotizing enterocolitis [5,6]. Alpha-tocopherol achieves peak plasma concentrations at 4–6 h after enteral administration and at 1 h after intravenous administration [1]. The half-life is not well characterized, and ranges from 2–20 h depending on isomer, population, and study; therefore, recommendations for twice-daily dosing are encouraged [1].

## 4. Mechanism of Action

### 4.1. Anti-Oxidative Effects of Vit E

**Figure 1:**
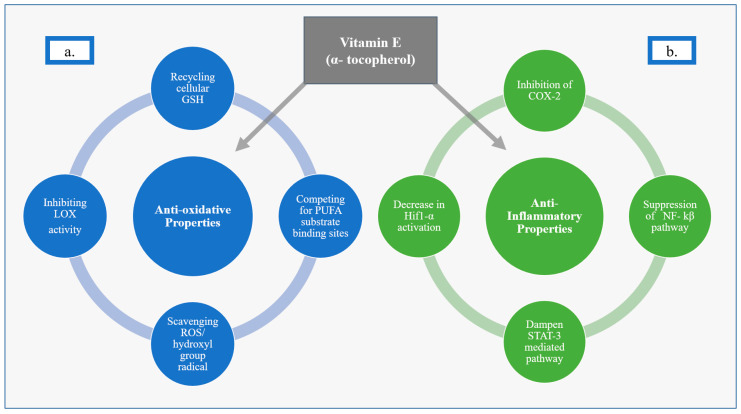
Anti-Oxidative and Anti-Inflammatory Properties of Vitamin E (α-tocopherol) (**a**) Anti-oxidative Properties of Vit E. (**b**) Anti-inflammatory Properties of Vit E.

Vit E, as α-tocopherol, has been described as having anti-oxidative properties that include recycling intracellular glutathione (GSH), scavenging reactive oxygen species (ROS), inhibiting lipoxygenases (LOX), competing for polyunsaturated fatty acid (PUFA) substrate binding sites, and scavenging hydroxyl group radicals. Much of Vit E’s protective effect is thought to be as a result of inhibiting oxytosis-ROS-dependent cell death via a lipophilic antioxidant effect on LOX. LOX enzymes are nonheme, iron-containing enzymes that catalyze deoxygenation of PUFAs [20,21]. Vit E inhibits LOX activity, which can otherwise trigger oxytosis, by competing at the substrate-binding site and by scavenging hydroxyl group radicals [20]. In addition, Vit E acts as a radical-trapping antioxidant (RTA) molecule that stops the autoxidation of chain-propagating peroxyl radicals, further aiding in its anti-oxidative properties [21,22]. Vit E is often described as an inhibitor of ferroptosis, though this is generally thought to just be an iron-dependent subset of oxytosis [20,21,23,24,25]. Similar to oxytosis more broadly, ferroptosis is also associated with accumulation of lipid ROS and depletion of plasma membrane PUFAs. Therefore, Vit E is thought to help stabilize and protect cell membranes [21,26]. Vit E’s ability to protect cells against oxytosis-driven cell death has been well documented and may be secondary to this protection of the integrity of cellular membranes from oxidative stress [24,25,27] (Figure 1).

### 4.2. Anti-Inflammatory Effects of Vit E

Vit E has also been described as a suppressor of pro-inflammatory pathways [1,20,21,22,28,29]. The anti-inflammatory properties of Vit E are thought to be secondary to inhibition of the secretion of inflammation-mediating molecules, such as interleukins 1 and 8, which are produced by the action of enzymes such as cyclooxygenase-2 enzyme (COX-2) [22,29]. Vit E also acts via suppression of pro-inflammatory signals downstream of nuclear factor kappa beta (NF-kβ) and signal transducer and activator of transcription 3 (STAT-3)-mediated pathways [28,30], as well as inhibiting the activation of hypoxia-inducible factor 1 alpha (Hif1 α) [28,31] (Figure 1).

## 5. Neuroprotection in Preclinical Models

**Figure 2:**
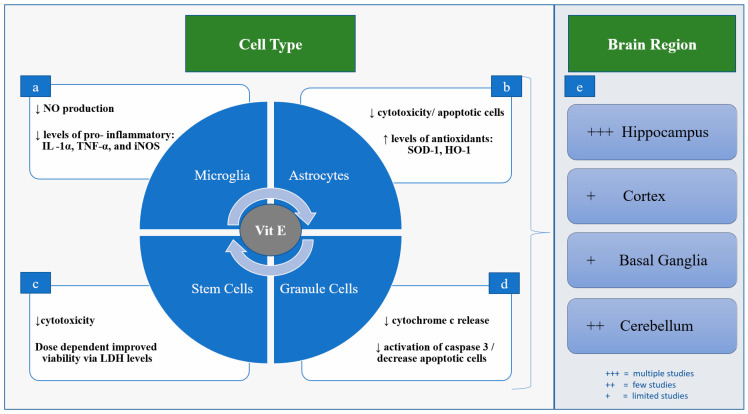
Proposed Neuroprotective Mechanisms of Vit E. (**a**) In mice and rat microglia, Vit E dampens activation via decreased production of NO, IL-1α, TNF-α, and expression of iNOS. (**b**) In rat astrocytes, Vit E improves cell viability and increases levels of antioxidant enzymes heme oxygenase (HO)-1 and superoxide dismutase (SOD)-1. (**c**) In rat mesenchymal stem cells (MSCs) Vit E improves cell viability, decreasing LDH release in a dose-dependent manner. (**d**) In rat cerebellar granule cells Vit E inhibits cytochrome c release and decreases activation of caspase 3, resulting in fewer apoptotic cells. (**e**) Vit E’s role in neuroprotection has been examined most often in the hippocampus and cerebellum, with fewer studies looking at the cortex and basal ganglia.

Preclinical studies to date have largely used various rodent in vitro and in vivo models of brain injury, where Vit E has been shown to decrease both inflammation and oxidative stress, with resulting neuroprotection [22,32,33,34,35,36,37]. These effects have been examined most frequently in the hippocampus and cerebellum, with fewer studies looking specifically at other regions such as the cortex and basal ganglia [7,38,39] (Figure 2). For example, Vit E deficiency in rodents has been shown to result in axonal degeneration in the hippocampus [38]. A recent study that used pentylenetetrazole, a GABA-A receptor antagonist, to induce seizures in rats, demonstrated that Vit E (200 mg/kg) was able to reduce neuronal ferroptosis by inhibiting LOX expression in the hippocampus [40]. Similarly, Vit E has been shown to improve cell viability in glutamate-induced ferroptosis of HT22 hippocampal cells [41]. In cultured astrocytes derived from the brains of newborn rats and then exposed hypoxic ischemic injury with oxygen glucose deprivation (OGD), Vit E (10 μg/mL) improves cell viability, decreasing both LDH release and number of apoptotic cells [42]. Pretreatment with Vit E in the astrocyte OGD model resulted in increased antioxidant capacity demonstrated through higher levels of heme oxygenase (HO)-1 and superoxide dismutase (SOD)-1); however, it did not significantly reduce interleukin (IL)-6, IL-1β, or tumor necrosis factor (TNF)-α [42]. Studies have also shown that inhibition of GSH recycling with the Xc-inhibitor erastin results in uncontrolled lipid peroxidation, which can be either masked or rescued in part by Vit E [25,43]. In a ferret organotypic brain slice model, Vit E (25 IU/kg) administered after OGD decreased cytotoxicity and inhibited oxidative stress by maintaining higher levels of GSH, and decreased transcription of genes responsible for oxidative stress and inflammation [7]. Vit E is also thought to dampen microglia activation in both mice and rats via decreased production of proinflammatory nitric oxide (NO), IL-1α, TNF-α, and reduce the expression of inducible nitric oxide (iNOS), though this effect has predominately been described in the hippocampus [2]. In rat cerebellar granule cells exposed to the apoptosis-inducer 1-methyl-4-phenylpyridinium, Vit E has been shown to inhibit cytochrome c release and decrease activation of caspase 3, resulting in fewer apoptotic cells [39] (Figure 2).

Additional evidence for the mechanisms of action of Vit E is provided by model systems outside of the brain. For instance, pretreatment of rat mesenchymal stem cells (MSCs) with Vit E (50–100 μM) prior to hydrogen peroxide-induced oxidative stress resulted in improved viability of cells and decreased LDH release in a dose-dependent manner [44]. In hemopoietic cells from mice who underwent ablation of glutathione peroxidase 4 (GPX4), perturbated reticulocyte maturation from uncontrolled lipid peroxidation was seen, which was reversed with supplementation of dietary Vit E (55 ppm) [45]. GPX4 is an enzyme that uses reduced glutathione as a substrate to scavenge lipid radicals [45]. In cultured Pfa-1 fibroblasts, ferroptosis induced either by erastin or GPX4 depletion was rescued with α-tocopherol (1 μM) to similar levels of cell viability as control cells after 24 h [43]. Vit E (50 μM) has also been shown to inhibit microglial after stimulation with LPS, decreasing the activity of NF-kβ and thereby decreasing iNOS expression and cytokine responses [46] (Figure 2).

## 6. Neuroprotection in Neonates

### 6.1. Role Prenatally

**Figure 3:**
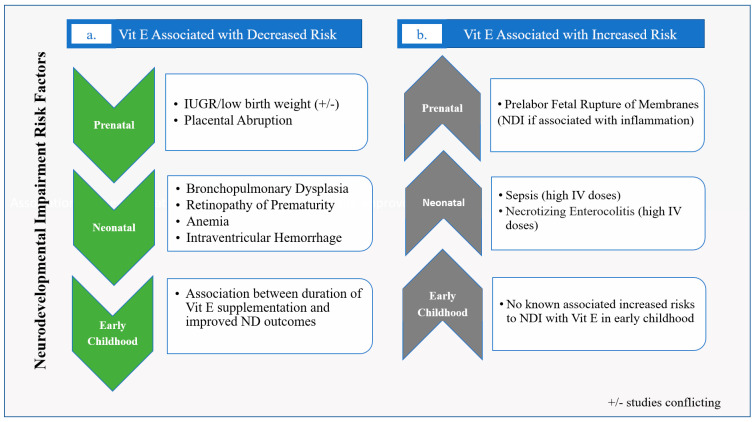
Clinical Studies of Vitamin E in Neonates for results where (**a**) Vit E is associated with a decreased risk of factors that are associated with NDI, and (**b**) Vit E is associated with an increased risk of factors that are associated with NDI.

Vit E has been shown to be involved in implantation, development of the embryo, maturation of the placenta, and protection of the fetus against oxidative stress [11]. There is low placental transfer of Vit E, though the naturally occurring RRR- α-tocopherol form has been shown to move more readily across the placenta than the racemic α-tocopherol synthetic form [47]. When looking at Vit E supplementation during pregnancy, a 2008 Cochrane review found no benefit in mothers supplemented with antioxidant treatments, including Vit E, during pregnancy for incidence of prematurity, small for gestational age (SGA) birth, or pre-eclampsia compared to placebo; however, Vit E was not studied alone nor was it part of every regimen included in the meta-analysis [48]. A Cochrane review in 2015 examining the effect of Vit E supplementation in pregnancy on perinatal events found no benefit, except for decreased placental abruption. This analysis also found that Vit E appeared to increase pre-labor fetal rupture of membranes [49], which itself has been associated with neurodevelopmental impairment (NDI) [50] (Figure 3). However, subsequent smaller individual studies examining growth and Vit E have found that higher maternal levels of Vit E are associated with improved fetal growth [51], and low Vit E levels have been associated with SGA birth [52] (Figure 3). If Vit E does improve fetal growth, one major presumed mechanism would be that higher Vit E levels improve the ability to mitigate inflammatory and oxidative stressors in utero; however, better overall nutritional status may also play a role [51,52]. Since fetal growth restriction, which is associated with low Vit E levels, is a known risk for NDI [53,54], maintaining adequate maternal Vit E levels appears prudent in order to optimize neurodevelopmental potential.

### 6.2. Role in Preterm Neonates

In premature infants, Vit E mitigates the downstream effects of oxidative stress and inflammation in multiple disease processes [5,6,37,55,56,57]. Clinical data has shown that Vit E has varying degrees of benefit in parenteral nurtrition associated liver disease (PNALD), retinopathy of prematurity (ROP), intraventricular hemorrhage (IVH), and bronchopulmonary dysplasia (BPD) [5,6,55,56,57], the latter three being tied to later neurodevelopmental outcomes (Figure 3). More specifically, clinically attenuated inflammatory responses are associated with higher α-tocopherol levels. In one study, 60 preterm neonates (gestational age 26–32 weeks) were randomized to receive either medium-chain triglyceride ω-3 polyunsaturated fatty acid (MCT/ω-3), PUFA-enriched intravenous fat emulsion (IVFE) (intervention group), or soybean oil-based IVFE (control group) [37]. The α-tocopherol content in the PUFA-enriched IVFE was 200 mg/L, whereas the α-tocopherol of the soybean oil-based IVFE was 38 mg/L [37]. Samples taken at consecutive timepoints demonstrated significantly lower levels of the pro-inflammatory cytokines IL-6 and IL-8 in the intervention group at 15 days and 30 days after birth, with concurrently higher α-tocopherol levels [37]. As PUFA-enriched IVFE has been reported to be associated with attenuated inflammatory markers in preterm neonates compared to Vit E-deficient soybean oil-based lipids, Vit E may therefore play a crucial role as part of this effect [37]. In a randomized double-blind, parallel-group study in preterm neonates <1500 g and <32 weeks, higher antioxidant markers and higher Vit E levels were found in infants given a combination lipid emulsion of soybean oil, medium-chain triglycerides, olive oil, and fish oil (SMOF) compared to standard fat emulsion intralipids. Specifically, 38 infants were studied and those that received SMOF lipid fat emulsion compared to standard fat emulsion had significantly higher levels of Vit E and total antioxidant potential (TAP) when measured at consecutive time points (day 0, 7, 14, and at discharge) [36]. These findings again give plausible support for the role of Vit E role in lowering oxidative stress and inflammation. Furthermore, since inflammation and oxidative stress markers have been shown to inversely correlate with NDI [58], Vit E’s role as a neuroprotective agent may mitigate these stressors. For example, oxidative stress and inflammation have been shown to affect vulnerable cell populations in the neonatal brain, including the subplate neurons and oligodendrocyte precursors involved in early development, perhaps becoming a target for Vit E [59].

### 6.3. Role in Term Neonates

Vit E is critical for CNS function beyond the gestational period of development, and its deficiency has been linked to neurodevelopmental delay [11]. Vit E deficiency in term infants as well as into childhood can lead to progressive neurological disorders, including sensory deficits and hyporeflexia in addition to spinocerebellar ataxia [11,60,61]. The above-mentioned anti-oxidative and anti-inflammatory properties of Vit E may provide neuroprotection both postnatally and after hypoxic ischemic injury [62]. One of the most devastating neurological injuries in term infants is hypoxic ischemic encephalopathy (HIE), secondary to perinatal asphyxia, where inflammation and oxidative stress are significant components of the pathogenesis [3]. As the brain continues to mature, neurogenesis and angiogenesis respond to ischemic changes differently [59], thus dampening those effects may give a plausible mechanism for neuroprotection with Vit E in term infants. Although this has not been assessed clinically, term-equivalent studies such as those performed in ferret organotypic brain slices exposed to OGD suggest that Vit E is neuroprotective after hypoxic-ischemic injury in the term-equivalent brain [7].

## 7. Vit E Supplementation in Neonates

**Figure 4:**
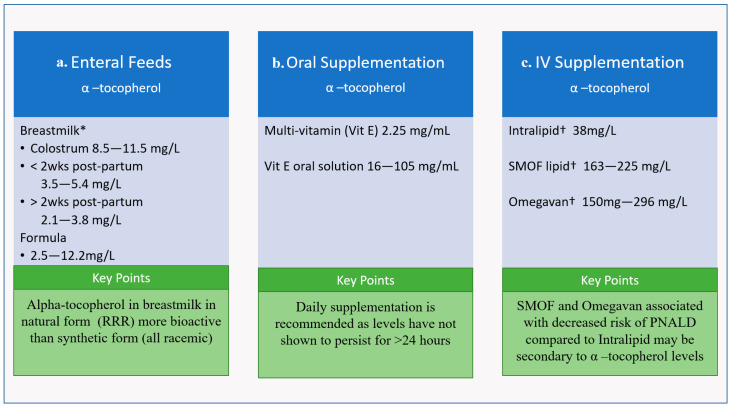
Concentration of alpha-Tocopherol (active form of Vit E) (**a**) enteral feeds, (**b**) oral supplementation, and (**c**) IV supplementation in common neonatal formulations and key points regarding each supplement category. * Pasteurization does not diminish Vit E levels † Manufacturer Fresenius Kabi (Uppsala, Sweden).

Postnatal Vit E supplementation is vital for healthy CNS development in newborns since only small amounts are thought to accrue in utero [47]. Vit E is considered safe in preterm and term infants at serum concentrations of 8.5–59 μmol/L (0.5–3.5 mg/L) [56]. A recent 2022 systematic review found that α-tocopherol levels ranged from 3.9 to 8.5 μmol/L in the preterm infant and 4.9 to 14.9 μmol/L in the term infant [11]. Although there is no certainty as to what should be considered Vit E deficiency, most studies agree that α-tocopherol levels <12 μmol/L are inadequate. As a result, depending on the study, 19% to 100% of newborns may have insufficient levels of Vit E [11,63]. Since Vit E already has a known safety profile and is a component of neonatal intravenous lipids and routine neonatal oral supplementation [6,56,64] (Figure 4), it could be an ideal pharmacological agent to promote neuroprotection in neonates if it is proven to be neuroprotective in humans, and the optimal dose, route, and concern regarding risks with high-doses in VLBWs is better delineated [65].

### 7.1. Vit E in Human Milk and Formula

Vit E is a component of human milk, and the level of Vit E in human milk has been shown to be decreased with maternal obesity, smoking, and preterm birth [63]. Importantly, pasteurization of donor breast milk does not alter its Vit E levels [66]. It is recommended that lactating mothers supplement with Vit E to obtain their daily allowance of 19 mg, though women with higher intakes of PUFAs have higher content of α-tocopherol in their breastmilk, which is consistent with literature around the linear relationship between Vit E and the oils, from which most dietary PUFAs are derived [63,67]. Alpha tocopherol levels in breastmilk vary over time; colostrum contains the most at approximately 8.5–11.5 mg/L, breastmilk one to two weeks postpartum contains 3.5 to 5.4 mg/L, and breastmilk more than 2 weeks postpartum contains only 2.1–3.8 mg/L. (Figure 4) [10,63]. Interestingly, hindmilk has been shown to have higher Vit E levels than foremilk. Maternal supplementation with Vit E 400 IU day in lactating mothers only made modest increases in levels in breastmilk, which may suggest a need for higher supplementation [63]. By comparison, the α-tocopherol content of infant formula ranges between 2.2 and 12.5 mg/L, with the upper end potentially ~10× fold greater compared to average breastmilk Vit E content (Figure 4) [63], the outcome of which is uncertain.

### 7.2. Vit E in Oral and Intravenous Supplementation

Current literature recommendations for α-tocopherol supplementation are 2.5–11 mg/kg/d for VLBWs and 2.5–3.5 mg/kg/d for term neonates [13,68]. Therefore, preterm neonates are frequently given oral or intravenous supplementation due to higher requirements. Infants on enteral feeds are supplemented with oral solution, either as a multi-vitamin that provides 2–3 mg/mL and/or different formulations of Vit E solution that provide 16–105 mg/mL to ensure adequate supply [11] (Figure 4). Vit E has been given to preterm neonates at doses up to 50 IU/kg (22.5 mg/kg); a study in 2013 gave a one-time 50 IU/kg dose of dl-α-tocopherol acetate enterally via nasogastric/orogastric tube at 4 h after birth and found increases in serum α-tocopherol levels within the outlined safety profile at 24 h, but these levels did not persist [6].

The Vit E level in intravenous supplementation depends on the amount of PUFAs in the fat emulsion, with α-tocopherol found in higher concentrations in SMOF lipids compared to soybean oil-derived Intralipid [56] (Figure 4). As noted above, preterm neonates given SMOF have been shown to have a more favorable cytokine profile [37]. Vit E in oil lipid emulsions have also been shown to reduce PNALD in preterm pigs; a recent study demonstrated that Vit E supplementation in intralipid prevented serum and liver increases in biliary and lipidemic markers of PNALD, which is a common side effect of parenteral nutrition [57]. Similarly, in pediatric clinical populations, SMOF has been shown to decrease the prevalence of intestinal failure-associated liver disease from 32% to 12% compared to Intralipid [69] (Figure 4).

## 8. Long Term Neurodevelopmental Outcomes

There is some data suggesting that Vit E status is tied to long-term neurodevelopmental outcomes [70]. Studies have shown that prolonged deficiency of Vit E is associated with cognitive impairment in early childhood and supplementation improved performance IQ in school-aged children born with extremely low birth weight (ELBW) [70,71]. In the latter study, 259 school-aged ELBW children were analyzed in three groups: those that received no Vit E supplementation, those with Vit E supplementation until <6 months of age, and those with Vit E supplementation for >6 months of age. Supplementation consisted of 20 mg/kg/day of racemic α-tocopherol starting at either 3 or 4 weeks after birth. They found that those supplemented for more than 6 months had the best outcome. Interestingly, multivariable regression analysis found that the association between duration of Vit E supplementation and IQ was dose-dependent [71] (Figure 3).

## 9. Gaps in Knowledge

The ability of Vit E to penetrate into the central nervous system (CNS) has been identified as a potential barrier for neuroprotection. Vit E is thought to cross into the CNS after peripheral transport by lipoprotiens, but is found at ~100× lower concentration in the brain compared to the blood [27]. Although it may have some effect on peripheral immune responses, the ability of exogenous Vit E to increase Vit E levels in the CNS is paramount for it to be effective as a neuoprotective agent. Therefore, determining the CNS penetration and safety of Vit E at doses targeted for neuroprotection remains of critical importance. Formal in vivo pharmacokinetic and dose–escalation studies would be required to determine an optimal dose and timing in different models—both preterm and term—since Vit E absorption and CNS penetration via the blood brain barrier has led to variability in CNS concentrations of Vit E [6,64]. Further factors that may affect CNS delivery include the requirement of adequate lipoproteins for Vit E to bind to in order to cross blood brain barrier, though this may be offset by an increase in blood brain barrier permeability in prematurity and after HI injury [6,72]. Overcoming barriers to Vit E uptake in the brain may also require a more selective synthetic transport.

The exact mechanism(s) by which Vit E may mitigate injury to certain brain regions or cell types has yet to be fully elucidated. Accumulation of Vit E in the cortex, hippocampus, basal ganglia and cerebellum has been described in murine and primate brains, suggesting Vit E may play a specific regional role in neuroprotection [27,39,73,74] and in the ferret Vit E improves cytotoxicity selectively in the hippocampus and cortex [7]. Further studies are needed to focus on Vit E effects by cell type to help delineate its potential neuroprotective effects, such as regional differences in treatment response to local microglial responses to injury [75]. This may be a plausible reason for why the hippocampus, one of the brain regions most densely populated with microglia [76], showed a significant decrease in cytotoxicity after Vit E exposure post-injury.

Interestingly, differences have been seen in animal models between males and females, with males appearing to be more responsive to the neuroprotective effects of Vit E compared to females. In the ferret model, Vit E more effectively improves cell death after hypoxic ischemic injury in whole organotypic slice culture in males greater than females [7]. This may be secondary to sex differences in cellular signaling pathways of neurological injury, where females are relatively more resilient to ROS-mediated injury due to higher antioxidant enzyme defense systems [77]. At higher doses, antioxidants (including Vit E) can become pro-oxidant, with saturation perhaps seen earlier in female with higher baseline antioxidant defenses [77,78,79,80]. This specific vulnerability of males to oxidative stress suggests males may benefit to a greater extent from antioxidant treatments, such as Vit E, following neurological injury.

In summary, although Vit E is a promising neuroprotective agent for newborn infants, further studies will be critical to the translation of Vit E. Scenario-specific pharmacokinetic, dose–response, and safety studies are required to better understand optimal dosing windows and confirm any sex-dependence or off-target effect before clinical trials can be safely implemented.

## 10. Conclusions

Although many studies suggest that Vit E has a range of promising anti-inflammatory and anti-oxidative effects, it is not known whether these properties are effective in promoting clinical neuroprotection. In a recent review published in 2019, the authors concluded there is no data to support use of Vit E for perinatal neuroprotection; however, the authors cite a Cochrane Review from 2003 [3]. In the past two decades, new emerging studies on Vit E have provided a number of beneficial results, though evidence of neuroprotection is still largely limited to preclinical models. Recent adult studies have also shown promise in Vit E’s ability to mitigate inflammation and oxidative stress, further imploring the question of whether neonatal populations would also benefit [81]. The use of Vit E at enterally at current routine doses appears safe in both term and preterm infants; however high dose intravenous in VLBWs would not be recommended. The unique regional and sex-based properties of Vit E remain to be fully elucidated but may be exploited to target specific populations or patterns of brain injury that could benefit from it. Answering some of these questions in clinically relevant animal models may help to delineate a target that can be implemented in human randomized clinical trials. Additional clinical studies are also needed to decipher whether Vit E levels in neonates correlate with decreasing risk of NDI.

In summary, if Vit E is used via the appropriate route and dose, and an appropriate population is targeted to exploit its beneficial properties, it could serve as a safe neuroprotective agent in newborn infants, either alone or in combination with other therapeutics.

## Data Availability

Not applicable.
